# A randomized controlled trial on maximal strength training in 60 patients undergoing total hip arthroplasty: Implementing maximal strength training into clinical practice

**DOI:** 10.1080/17453674.2018.1441362

**Published:** 2018-03-01

**Authors:** Siri B Winther, Olav A Foss, Otto S Husby, Tina S Wik, Jomar Klaksvik, Vigdis S Husby

**Affiliations:** 1 Orthopaedic Research Centre, Department of Orthopaedic Surgery, Clinic of Orthopaedics, Rheumatology and Dermatology, St Olavs Hospital HF, Trondheim; 2 Department of Neuromedicine and Movement Science, Faculty of Medicine and Health Science, Norwegian University of Science and Technology NTNU, Trondheim; 3 Department of Mental Health, Faculty of Medicine and Health Science, Norwegian University of Science and Technology NTNU, Postbox 8905 MTFS, NO-7491, Trondheim, Norway

## Abstract

**Background and purpose:**

Total hip arthroplasty (THA) patients have reduced muscle strength after rehabilitation. In a previous efficacy trial, 4 weeks’ early supervised maximal strength training (MST) increased muscle strength in unilateral THA patients <65 years. We have now evaluated muscle strength in an MST and in a conventional physiotherapy (CP) group after rehabilitation in regular clinical practice.

**Patients and methods:**

60 primary THA patients were randomized to MST or CP between August 2015 and February 2016. The MST group trained at 85–90% of their maximal capacity in leg press and abduction of the operated leg (4 × 5 repetitions), 3 times a week at a municipal physiotherapy institute up to 3 months postoperatively. The CP group followed a training program designed by their respective physiotherapist, mainly exercises performed with low or no external loads. Patients were tested pre- 3, 6, and 12 months postoperatively. Primary outcomes were abduction and leg press strength at 3 months. Other parameters evaluated were pain, 6-min walk test, Harris Hip Score (HHS) and Hip disability and Osteoarthritis Outcome Score (HOOS) Physical Function Short-form score.

**Results:**

27 patients in each group completed the intervention. MST patients were substantially stronger in leg press and abduction than CP patients 3 (43 kg and 3 kg respectively) and 6 months (30 kg and 3 kg respectively) postoperatively (p ≤ 0.002). 1 year postoperatively, no intergroup differences were found. No other statistically significant intergroup differences were found.

**Interpretation:**

MST increases muscle strength more than CP in THA patients up to 6 months postoperatively, after 3 months’ rehabilitation in clinical practice. It was well tolerated by the THA patients and seems feasible to conduct within regular clinical practice.

Patients with osteoarthritis have reduced muscle strength in the affected limb preoperatively (Rasch et al. 2007), and muscle strength decreases further in the first postoperative week after total hip arthroplasty (THA) (Winther et al. 2016). The muscle strength is still reduced following the initial few weeks post THA (Holm et al. 2013, Winther et al. 2016). Reduced muscle mass, strength, and functionality seem to persist after completed rehabilitation (Reardon et al. 2001, Bertocci et al. 2004, Judd et al. 2014), in some studies up to years after surgery (Sicard-Rosenbaum et al. 2002, Rasch et al. 2010). Leg muscle strength affects ambulatory status following THA (Nankaku et al. 2014), and patients with the best muscle strength have higher physical function, quality of life scores, and lower pain levels (Rosenlund et al. 2016). Muscle strength is related to beneficial effects on functional performance such as chair raising, stair climbing, and gait performance (Samuel et al. 2012, Buirs et al. 2016, Unhjem et al. 2017), and seems important for minimizing postoperative limping (Horstmann et al. 2013).

The prolonged deficits in muscle strength of the operated leg after THA indicate a potential for improvements in postoperative care (Judd et al. 2014). It is recommended that persistent asymmetries in hip flexor muscles should receive focused attention during rehabilitation (Friesenbichler et al. 2017) and that muscle-strengthening exercises should be continued for at least 1 year after THA (Shih et al. 1994). Although muscle strength of the operated leg is considered an important postoperative outcome, what specifically constitutes the optimal rehabilitation program after THA remains unclear (Westby et al. 2014). Conventional physiotherapy (CP) consisting of exercises with low or no external load is frequently used in rehabilitation after THA; however, the effect has been questioned (Minns Lowe et al. 2009, Husted 2012). Physiotherapist-directed rehabilitation appears similarly effective performed unsupervised at home compared with supervised by a physiotherapist in an outpatient setting (Coulter et al. 2013). Reasonably there is great potential for improvement as weight-bearing exercises and progressive strength training have shown favorable outcomes (Di Monaco and Castiglioni 2013, Skoffer et al. 2015).

In a Fast-track perspective, it is suggested that studies should focus on early, intense postoperative strengthening (Rasch et al. 2010, Husted 2012, Holm et al. 2013, Judd et al. 2014). Immediate full weight bearing after THA is safe (Wolf et al. 2010), and strength exercises do not exacerbate postoperative pain (Mikkelsen et al. 2017). This enables patients to start their rehabilitation within the first week after surgery. In a previous efficacy study, patients <65 years who underwent unilateral THA following a 4-week early maximal strength training (MST) program under supervision from an exercise physiologist increased leg muscle strength more than CP after THA (Husby et al. 2009). In the present study, we aimed to evaluate the effect from 2 rehabilitation programs, in regular clinical practice. The primary outcome was muscle strength at 3 months’ follow-up. The null hypothesis is that there will be no intergroup differences.

## Patients and methods

The study was a prospective randomized controlled superiority trial with THA patients operated on using the posterior approach. All patients followed the standardized Fast-track course (Winther et al. 2015). Patients were randomly assigned receiving different postoperative rehabilitation at municipal physiotherapy institutes—either MST or CP—and logged their physical activity in a training diary up to the 6-monthly follow-up. The primary outcome was 1RM in abduction and leg press strength, a surrogate outcome measure related to functional performance. Secondary outcomes were pain assessed by the numeric rating scale (NRS), distance covered during the 6-minute walk test (6MWT), Harris Hip Score (HHS) and Hip disability and Osteoarthritis Outcome Score Physical function Shortform score (HOOS-PS).

### Patients

**Figure 1.**
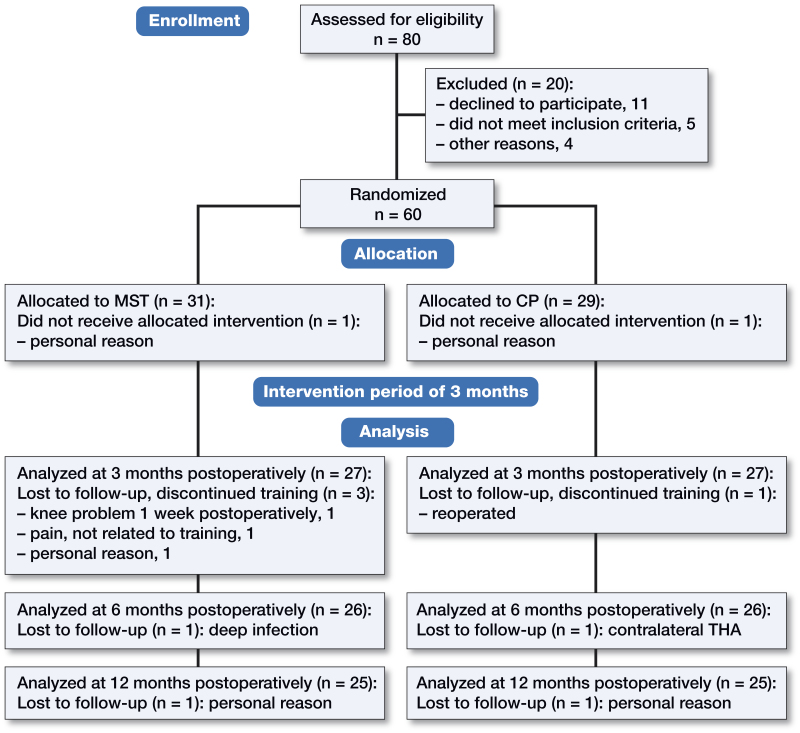
Patient inclusion and follow-up in the maximal strength training (MST) and conventional physiotherapy (CP) groups.

Patients diagnosed with primary osteoarthritis, scheduled for elective THA surgery at St Olavs University Hospital, Norway, living within short travel distance to the hospital, were asked to participate in the study by a nurse at the admission office, and assessed by an orthopedic surgeon. Exclusion criteria were severe osteoarthritis of the contralateral hip, not fully recovered from previous THA surgery, communication difficulties, discharged to a rehabilitation institute, or any illness or disorder that could influence the training and/or physical testing performance. Between August 2015 and February 2016, 80 patients were assessed for eligibility. Finally, 60 patients gave written consent to participate (Figure 1). The randomization was stratified by sex and concealed by using a web-based service provided by the research department at the university.

### Testing

**Figure 2.**
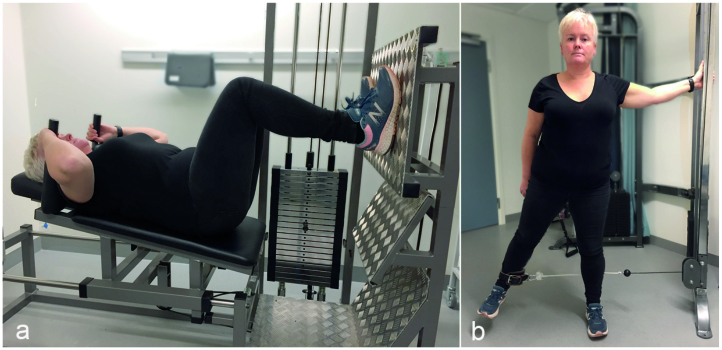
Set-up for the (a) leg press ergometer and (b) abduction pulling apparatus.

Patients were tested preoperatively, and at 3, 6, and 12 months postoperatively. 2 physiologists and 1 physiotherapist, all highly experienced with patient testing, conducted the physical tests starting with the 6MWT where the patient walked back and forth in a 50-meter hallway for 6 minutes. 1RM leg press was tested in an ergometer device (Steens Physical, Ring Mekanikk AS, Norway) (Figure 2a). 1RM abduction strength was tested in a pulling apparatus (Pivot 820, Sports Master, Norway) (Figure 2b). Pain was assessed by the NRS: “On a scale from 0 to 10, where 0 is no pain and 10 is the worst pain imaginable, can you define the pain you have right now?” The disease-specific questionnaires HHS and HOOS-PS were obtained preoperatively and at 3 and 12 months postoperatively. Maximum score for the HHS is 100 points. HOOS-PS is scored from 0 to 100 with zero being optimal.

### Intervention

In accordance with current practice, the patients contacted the preferred physiotherapy institute the day after discharge and scheduled the first appointment. The physiotherapy institutes prioritized these patients. Patients in the MST group could attend 1 out of 5 municipal physiotherapy institutes that had consented to participate in the study. The institutes received the training protocol and were instructed in what manner to supervise the patients performing the specific exercises: leg press and abduction strength of the operated leg. 1-week postoperatively, 1RM in leg press and abduction of the operated leg were tested at the hospital to decide the initial weight load for the patients during the first training session. Each training was provided individually and started with a preferable warm-up exercise followed by the 2 strength-training exercises. Patients performed 5 repetitions ×4 series, starting with a load equal to 85–90% of 1RM, with emphasis on maximal mobilization of force in the concentric part of the movement. The series were separated by 1- to 2-minute resting periods, and the load was increased when the patient could perform 6RM. MST patients were prescribed 3 weekly physiotherapy visits for 3 months in accordance with clinical practice, and the adherence was assessed by means of self-reported visits collected from the training diaries. If required, the patients could receive stretching guidance and treatment by the physiotherapist, but no additional strength training of the operated leg was offered. After the intervention period, training with the physiotherapist was optional up to 6 months.

Patients in the CP group were instructed to follow the conventional rehabilitation regimen advised by the hospital—outpatient physical therapy for 3–6 months—and to follow their physiotherapist’s guidance. They could choose any municipal institute within the municipality in which they resided. CP consisted of different type of exercises performed with low or no external loads (10–20 repetitions in each series). Warm-up exercises were mainly cycling, step and treadmill walking. Other exercises used were aquatic exercises, balance training, range-of-motion exercises, massage, and sling exercises.

### Group-size calculation

To reveal an effect size of approximately 1 based on the primary outcome—1RM in leg press strength—a between-group difference of 20 kg (SD 21) was considered the minimally clinically important difference and used for sample size calculation. With a significance level of 5% and a power of 90%, 24 participants were required for each group. 60 patients were included to account for dropouts.

### Statistics

**Figure 3.**
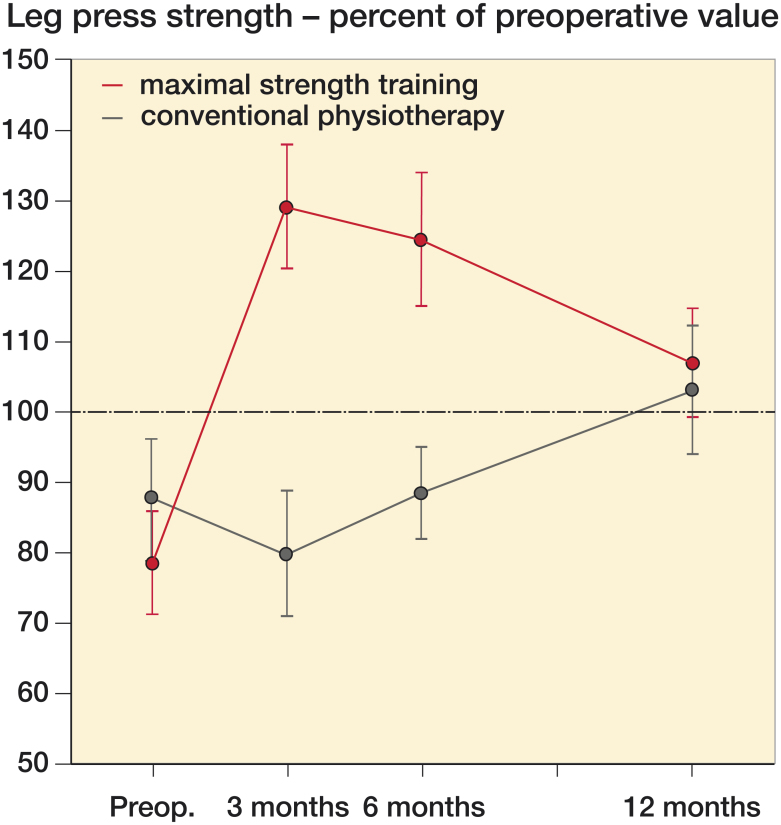
Leg press strength of the operated leg compared with preoperative values (100%) of the non-operated leg in the maximal strength training (MST) and conventional physiotherapy (CP) groups at 3, 6, and 12 months postoperatively. Model estimate with 95% confidence intervals.

**Figure 4.**
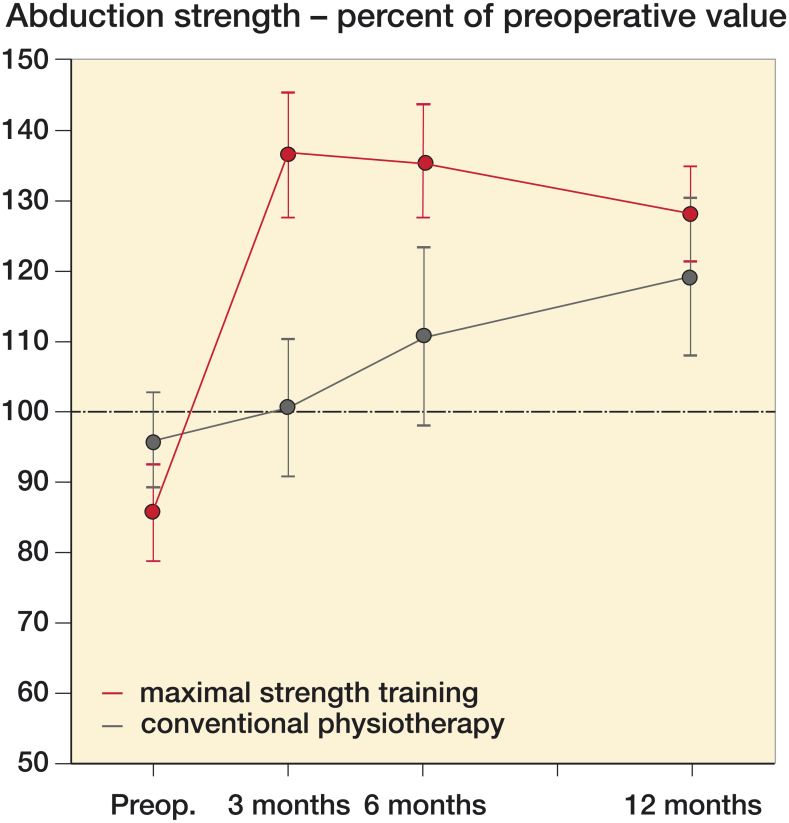
Abduction strength of the operated leg compared with preoperative values (100%) of the non-operated leg in the maximal strength training (MST) and conventional physiotherapy (CP) groups at 3, 6, and 12 months postoperatively. Model estimate with 95% confidence intervals.

**Figure 5.**
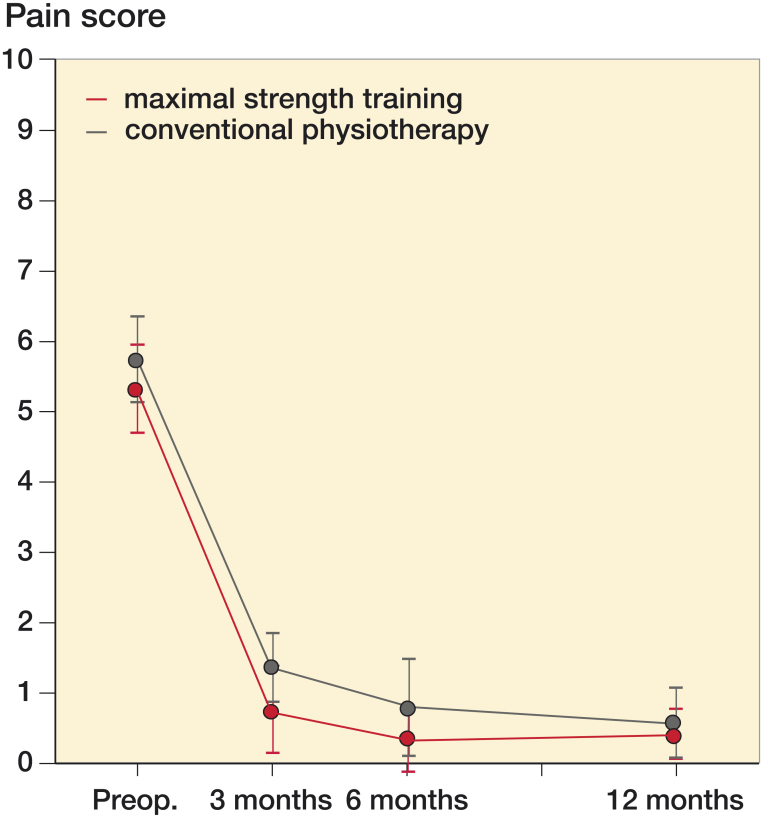
Pain score during mobilization on the numeric rating scale (NRS: 0–10) in the maximal strength training (MST) and conventional physiotherapy (CP) groups preoperatively and at 3, 6, and 12 months postoperatively. Model estimate with 95% confidence intervals.

A General Linear Mixed Model (GLMM) was used to analyze all outcome variables. Strength measures were expressed as percentage of the preoperative score from the non-operated leg. The measured values were used when analyzing the other outcome variables. The preoperative value of the tested variable was included as a covariate representing a baseline control as well as correcting for eventual initial imbalance between groups. The preoperative pain score was additionally included as a covariate in all analyses. The 2 groups and time points served as fixed factors in the analyses. Interaction terms were used to acquire detailed comparisons between groups and between time points. Robust estimation was chosen to handle violations of model assumptions. P-values were Bonferroni corrected to adjust for multiple comparisons. Normality of residuals was verified by histograms. The covariates included in the models were a priori selected based on clinical evaluation. The analysis was based on an intention-to-treat principle, but there were no patient crossovers between the two treatment groups. In total, 3.2% of all data points were missing from follow-ups. As the GLMM is robust in handling missing data, there were no data set imputations made. Statistical significant intergroup differences of both primary endpoints were required to reach a confirmatory conclusion. Figures 3–5 represent model estimates, adjusted for covariates. Statistical analyses were performed using the software package IBM SPSS Statistics for Windows (version 21.0; IBM Corp, Armonk, NY, USA).

### Ethics, registration, funding, and potential conflicts of interest

The study was approved by the regional ethics committee (2010/3373) and conducted in accordance with the Declaration of Helsinki. The study was registered at CilnicalTrials.gov (NCT02498093) and supported by the Liaison Committee between the Central Norway Regional Health Authority (RHA) and the Norwegian University of Science and Technology [grant number 2010/708/MOCA]. The funding sources had no impact on the analyses, interpretation, or presentation of the data.

## Results

Demographic variables of the patients are presented in Table 1.

**Table 1.** Preoperative values from patients in the maximal strength training (MST) and conventional physiotherapy (CP) group. Values are mean (SD) or (range)

	MST (n = 31)	CP (n = 29)
Sex (F/M)	17/14	15/14
Age	61 (35–77)	66 (44–83)
BMI	28 (4)	27 (3)
Leg press: operated leg (kg)	76 (29)	84 (33)
Abduction: operated leg (kg)	9 (3)	10 (4)
Pain mobilization (NRS)	5.3 (2.2)	5.8 (2.0)
6MWT (m)	499 (124)	498 (125)
HHS	62 (13)	62 (14)
HOOS-PS	36 (16)	35 (14)

NRS: Numeric rating scale (0–10; 0 is no pain, 10 is worst pain imaginable), 6MWT: 6-minute walk test, HHS: Harris Hip Score, HOOS-PS: Hip disability and Osteoarthritis Outcome Score Physical function Short-form score.

### Compliance

MST started 15 (SD 4) days after surgery. The average number of physiotherapy visits during the 3-month intervention period was 24 (4), and each training session was completed within 30 minutes. 6 patients in the MST group continued training with the physiotherapist from 3 to 6 months postoperatively, on an average of 2 (1) times per week, but did not perform the 1-leg MST.

CP started 19 (8) days after surgery. The average number of physiotherapy visits until 3 months was 19 (7), each lasting 55 (12) minutes. 12 patients in the CP group continued training with the physiotherapist from 3 to 6 months postoperatively, at an average of 2 (1) times per week.

### Maximal strength

Patients in the MST group were stronger in leg press strength of the operated leg than the CP group at the 3 and 6 months’ follow-up (p < 0.001). 1-year postoperatively, no statistically significant intergroup differences were found (p = 0.5) (Figure 3).

Patients in the MST group were stronger in abduction strength of the operated leg than the CP group at the 3 and 6 months’ follow-up (p ≤ 0.002). 1-year postoperatively, no statistically significant intergroup differences were found (p = 0.2) (Figure 4).

### Pain, 6MWT, HOOS-PS, and HHS

No statistically significant intergroup differences in pain score (p > 0.1) (Figure 5), 6MWT (p > 0.7), HOOS-PS (p > 0.6) or HHS (p > 0.3) were found at any follow-up (Table 2).

**Table 2.** Postoperative values from patients in the maximal strength training (MST) and conventional physiotherapy (CP) groups. Values are mean (SD)

	Leg press (kg)		Abduction (kg)		6MWT (m)		HHS		HOOS-PS	
Month	MST	CP	MST	CP	MST	CP	MST	CP	MST	CP
3	120 (31)	77 (30)	13 (4)	10 (4)	583 (97)	578 (129)	91 (14)	87 (12)	14 (12)	14 (13)
6	114 (23)	84 (22)	13 (3)	10 (4)	607 (94)	596 (111)				
12	101 (27)	100 (26)	12 (4)	12 (4)	627 (96)	628 (110)	95 (7)	93 (9)	8 (10)	8 (10)

6MWT; 6-minute walk test, HHS; Harris Hip Score, HOOS-PS; Hip disability and Osteoarthritis Outcome Score Physical function Short-form score.

## Discussion

Early postoperative MST increases muscle strength more than CP in THA patients within 3 months postoperatively. This strength difference persists up to 6 months postoperatively. No statistically significant intergroup differences were observed with respect to pain, 6MWT, HHS, or HOOS-PS. Husby et al. (2009) previously demonstrated that MST 5 times a week for 4 weeks, initiated 1 week postoperatively, increased strength more than CP in THA patients. Patients included in that study were restricted to unilateral osteoarthritis diagnosis and were relatively young, i.e., < 65 years. In contrast to our study, the training was located at a rehabilitation institute and closely supervised by 2 exercise physiologists during the entire intervention period. Our study was completed in accordance with current clinical practice as the patients had outpatient physiotherapy at municipal institutes, and few exclusion criteria. This demonstrates that MST can be implemented into clinical practice and the results generalized outside the experimental setting.

Muscle strength is considered an important outcome after primary THA (Westby et al. 2014). Consequently, we see this as a clinically meaningful surrogate measure. In a systematic review by Skoffer et al. (2015), weak evidence of a beneficial effect of progressive resistance training pre/post THA on muscle strength and functional capacity was found. Suetta et al. (2004) found that only 2 exercises with supervised progressive strength training (8–20 repetitions) of the operated leg increased muscle strength more than home-based training 12 weeks after THA. Conversely, Mikkelsen et al. (2014) did not find supervised progressive strength training twice a week superior to home-based exercise in improving muscle strength, 10 weeks after THA surgery. The training sessions in their study lasted longer than in our study, and consisted of 4 exercises with 10–12 repetitions. In the early postoperative phase, patients are physically reduced. Training should therefore be simple, task specific and focused on targeting the affected muscles (Rasch et al. 2010). In our study, each training session lasted about twice as long for patients in the CP compared with the MST group (55 vs. 30 minutes). Shorter training sessions may increase the performance during training.

Muscle strength in the MST group decreased when the training was terminated. Still, they were stronger than the CP group, and exceeded the preoperative values of the non-operated leg at all follow-ups. The gradually reduced muscle strength in the MST group after the training intervention might be expected as muscle strength has an expiration date and the benefits from the training cannot be stored for later. In the CP group, muscle strength continued to increase from 3 to 6 months and further up to the 1-year follow-up. Patients in the CP group did not reach the preoperative leg press values of the non-operated leg until the 1-year follow-up. Our results are consistent with previously reported findings of only modest strength improvements after CP (Minns Lowe et al. 2009), and the fact that strength increased after completed rehabilitation (Beaupre et al. 2014). It has therefore been suggested that THA patients should continue strength training of the operated hip for at least 1 year postoperatively or should be given a more advanced exercise program later in their recovery (Shih et al. 1994, Trudelle-Jackson et al. 2002). The MST group had on average a few more training sessions than the CP group during the intervention period, but each session lasted half the time so that the total volume was higher in the CP group. This demonstrates that performing MST in clinical practice requires less effort and resources than CR. Furthermore, only half as many patients in the MST group as the CP group preferred to continue training with the physiotherapist after the 3 months’ intervention period, due to subjective perception of equal bilateral leg strength and independence, and being competent to continue training on their own as reasons for terminating rehabilitation. The early substantial increase in muscle strength in the MST group suggests that 3 months’ rehabilitation might be sufficient when done efficiently.

Early postoperative rehabilitation should focus on preventing the great reduction in muscle strength demonstrated in these patients. Therefore, especially in the early phase when the total training volume ought not be too high, strength training should be a priority. As soon as leg muscle strength is regained, the patient’s endurance capacity and physical activity should be increased to prevent various lifestyle-related diseases. The MST and CP groups approach each other in muscle strength 1-year postoperatively with a decreasing/increasing trend respectively, with no statistical significant intergroup difference (Figure 3). A reasonable question is whether the patients are completely rehabilitated when compared with healthy age-matched controls 1 year postoperatively. Previous studies have shown that THA patients have less strength in the lower extremities than healthy age-matched controls 1 year postoperatively (Sicard-Rosenbaum et al. 2002, Judd et al. 2014). In the present study, the MST group probably could have maintained superior muscle strength, for example, by having 1 MST session a week (Lexell et al. 1995).

Both groups expressed very little pain at the 3-month follow-up with a mean NRS score of 0.8 in the MST and 1.4 in the CP group, which consistently decreased by the 1-year follow-up in both groups, with no statistically significant intergroup differences (Figure 5). This is an important finding, as heavy strength training early after THA is anticipated to induce more pain than exercising at lower intensities. Our results are in accordance with findings from Mikkelsen et al. (2017) who found that substantial load progression during strength training did not exacerbate postoperative pain after THA.

We did not find any statistically significant intergroup differences in 6MWT, HHS, or HOOS-PS. The 6MWT is foremost designed to evaluate cardiorespiratory fitness and does not appreciably challenge the leg muscle strength. The stair-climbing test could have differentiated between the groups as it evaluates tasks closely related to daily living situations/activities (Unver et al. 2015). All patients were treated according to the fast-track clinical pathway, previously shown to assign high scores for HHS and HOOS-PS (Winther et al. 2015). The ceiling effect of these scores might limit their validity for use in clinical trials and a more differentiating score, such as the forgotten joint score, could have been used.

The main limitation of our study is the lack of a healthy age-matched control group to identify the muscle strength of the normal population. This could have been informative when considering the results from the THA patients. Also, we did not record the patients’ exercise status from 6 to 12 months. The study was not designed with the power to evaluate the secondary endpoints, but they are interesting seen in context with the primary finding, and as descriptive information.

As muscle strength is important for physical function in both patients and healthy people (Nankaku et al. 2016, Unhjem et al. 2017), it is important to explore rehabilitation methods that can restore and improve muscle strength postoperatively (Westby et al. 2014). Further, the proportion of THA patients <70 years is increasing, and many are still employees and engaged in physical activities, demanding effective rehabilitation (Hobbs et al. 2011, Smith et al. 2015). As a result, patients might be able to return faster to physical activities and work. For future studies, it might be beneficial to train these patients both pre- and postoperatively, and in combination with endurance training, since most patients experience a period of inactivity before surgery due to activity-related pain and contracture of the hip.

In summary, MST increases muscle strength more than CP in THA patients after rehabilitation in clinical practice. The muscle strength difference persists up to 6 months postoperatively; however, the groups are approaching each other. 1 year postoperatively, no intergroup differences were found. MST is feasible to conduct in regular clinical practice and the results can be generalized to a wide THA population.

All authors contributed substantially to interpreting the results, and drafting and revising the article. SBW, VSH, OSH, TSW and OAF contributed to the study design; SBW and VSH collected data; OAF and JK conducted the statistical analysis; OSH and TSW screened the patients.

The authors would like to thank the physiotherapy institutes participating; Moholt physiotherapy, Tiller physiotherapy and manual therapy, Leangen physiotherapy, Frisk physiotherapy and Orkanger physiotherapy and osteopathy. They also thank physiotherapist Sølvi Liabakk-Selli for contribution in patient testing, and nurses Ann Kristin Green-Helgetun, Ann-Merete Kjøsnes, and Elin Ida Sletner for contribution in patient recruiting and coordination.

*Acta* thanks Thomas Bandholm and Henrik Husted for help with peer review of this study.
